# Monocytes as an Early Predictor for Patients with Acute Paraquat Poisoning: A Retrospective Analysis

**DOI:** 10.1155/2019/6360459

**Published:** 2019-07-15

**Authors:** Yong Zhao, Ya Qi Song, Jie Gao, Shun Yi Feng, Yong Li

**Affiliations:** Emergency Department, Cangzhou Central Hospital, No. 16 Xinhua Road, Yunhe Qu, Cangzhou City 061000, China

## Abstract

**Background:**

The predictive values of monocytes in the prognosis of patients with acute paraquat (PQ) poisoning are unclear. This retrospective study investigated the predictive values of monocytes in the prognosis of patients with acute PQ poisoning.

**Methods:**

Adult patients who suffered from acute PQ poisoning in the emergency care unit of Cangzhou Central Hospital from May 2012 to December 2018 were enrolled. The patients were divided into groups, namely, survival and nonsurvival, according to a 90-day prognosis. Moreover, correlation, logistic regression, receiver-operator characteristic (ROC), and Kaplan–Meier curve analyses were applied to evaluate the monocyte values used to predict the prognosis of patients with acute PQ poisoning.

**Result:**

Among the 109 patients, 45 survived within 90 days after the poisoning, resulting in a 41.28% survival rate. The monocyte count of the nonsurvivors was significantly higher than that of the survivors (*P*< 0.001). Correlation analysis showed that monocyte count positively correlated with plasma PQ concentration (r= 0.413;* P*< 0.001) and negatively correlated with survival time (r= 0.512;* P*< 0.001) and 90-day survival (r= 0.503;* P*< 0.001). Logistic regression analysis showed that elevated monocytes were the independent risk factors for the 90-day survival. The area under the ROC curve of the monocyte count used to predict the 90-day survival was 0.826 (95% CI: 0.751–0.904), the optimal cut-off was 0.51×10^9^/L, sensitivity was 73.4%, and specificity was 86.7%.

**Conclusion:**

This study demonstrated that elevated monocyte count is a useful early predictor of 90-day survival in patients with acute PQ poisoning. However, further studies are warranted to draw firm conclusions.

## 1. Introduction

Paraquat (PQ) is a nonselective contact herbicide that has long been used worldwide, especially in developing agricultural countries, such as China. PQ is highly toxic to humans, and ingestion of >15–30 mL of 20% (w/v) PQ can be fatal in humans [1]. PQ poisoning management has remained predominantly supportive, and the results of PQ poisoning treatment, including immunosuppressive therapy [2], prolonged extracorporeal elimination [3], and lung transplantation [4], have been disappointing [5]. Thus, the identification of accurate prognostic indicators is crucial.

Many prognostic indicators have been investigated to evaluate the prognosis of PQ poisoning. Plasma PQ concentration is a marker of severity and prognosis with acceptable sensitivity and specificity [6–10]. However, the measurement of plasma PQ concentrations requires extremely expensive, technical, and accurate equipment that may not be readily available in most hospitals. Prediction of the prognosis of the condition on the basis of clinical signs and symptoms has yet to be realized; the onset of symptoms after acute PQ poisoning requires time, and by then, the poisoning can be irreversible or fatal. PQ poisoning mortality rate is highly correlated with PQ amount intake. However, precisely identifying the amount of PQ through subjective descriptions is difficult. The area of ground glass opacities [11] and the ratio of injured lung volume fraction [12] displayed by high-resolution computed tomography images 4–6 days after intoxication can be used to evaluate PQ-induced pulmonary injury and clinical prognosis. Therefore, this approach is unsuitable for the early assessment of prognosis. Pulmonary radioisotope scanning may also predict the prognosis of PQ poisoning. However, its application is difficult [13]. Some studies suggested two severity scores of disease classification systems, namely, the acute physiology and chronic health evaluation II and sequential organ failure assessment scores, as acceptable predictors of acute PQ poisoning prognosis. However, their application to the early course of acute PQ poisoning is limited because of the complex scoring parameters and the need to record the worst value of each parameter within 24 h of admission [14, 15].

Recently, many studies have indicated that elevated monocyte count is closely associated with poor prognosis in patients with osteoarticular brucellosis [16], end-stage renal disease [17], gastric cancer [18], colon cancer [19], and carbon monoxide poisoning [20] and that they might be easily available and reliable prognostic biomarkers. Previous studies showed increases in monocytes through a blood test and destruction of alveolar structure [21, 22]. However, no previous studies investigated the association between monocytes and acute PQ poisoning prognosis. Therefore, in the current study, we determine whether monocytes may serve as an early predictor for patients who suffer from acute PQ poisoning.

## 2. Methods

### 2.1. Ethics, Consent, and Setting

This retrospective clinical study was conducted in accordance with the ethical guidelines of the 1975 Declaration of Helsinki and was approved by the Ethics Committee of the Cangzhou Central Hospital (No. 2017-090-01). However, informed consent was unavailable because of its retrospective nature. Informed consent regarding the treatment risk following acute PQ poisoning was obtained from all patients upon their initial admission. All data were gathered in the context of standard practice from clinical patient records without the need for informed consent. Then, the data were anonymized and stored in a protected database.

### 2.2. Patients

The medical records of patients with PQ poisoning who were already discharged from the hospital or died between May 2012 and December 2018 were collected for this retrospective analysis. The inclusion criteria were as follows: patients diagnosed with acute PQ poisoning by checking their plasma PQ concentrations; patients aged > 14; patients who suffered from PQ poisoning through oral intake; and hospital admissions within 12 h of poisoning. The exclusion criteria were as follows: patients who suffered from other pesticide poisoning, pregnant patients, cases with infection, cases with immunosuppressive therapy, or cases with blood systemic diseases.

### 2.3. Treatment Protocols

All patients were administered 1 g/kg activated carbon tablets plus Fuller's earth added to 250 mL of 20% mannitol via a gastric tube to minimize further absorption following gastric lavage with room warm water (≥5 L) [3]. All patients received one to three courses of 3 h active charcoal containing hemoperfusion (HP) therapy based on the result of urine PQ detection and clinical condition. Hemofiltration was performed when acute renal failure occurred. In addition, all patients received a unified therapeutic regimen including fluid infusion and diuresis (furosemide 40 mg/day for 3–5 days), immunosuppressant (methylprednisolone 0.375–1 * *mg/kg/day for 3 days), and antioxidants (vitamin C 3.0 g/day for 10–14 days, glutathione 2.4 g/day for 10–14 days).

### 2.4. Data Collection

At baseline, clinical data, including age, gender, time from ingestion to gastric lavage, alanine aminotransferase (ALT), creatinine, alveolar oxygen partial pressure (PaO_2_), plasma PQ concentration, and monocytes, were obtained. In consideration of the possible influence of HP and drug therapy on the predictive value of monocytes, blood samples obtained before HP and drug therapy were eligible, and only this single point of monocytes was used to assess the predictive value of monocytes. Elevated monocytes correspond to a level >1.0×10^9^/L (normal range: 0.2–1.0* *×10^9^/L). According to previous reports, patients with PQ poisoning usually die within several weeks after PQ ingestion. Thus, the study outcome was defined as 90-day survival, and the survival time was identified on the basis of medical records or telephone follow-up.

### 2.5. Statistical Analysis

All analyses were conducted using SPSS software version 13.0 (SPSS Inc., Chicago, IL). The independent sample t-test was used to evaluate measurement data if normal distribution was followed, and data were presented as mean ± standard deviation; otherwise, two-independent sample nonparametric tests were performed, and data were presented as median ± interquartile range. Categorical variables were analyzed using *χ*2 test or Fisher exact test. The Spearman rank method provides a nonparametric measure of correlation between two variables [23]. Factors with a value of* P*< 0* *.05 in the univariate analysis were further analyzed using a multivariate Cox proportional hazard model. A continuous covariate into a binary one when no cut-off point was established (previous published results or biological knowledge) was performed by using an outcome-oriented statistical method (such as the optimal cut-point estimation) [24]. A receiver-operator characteristic analysis was used to identify the cut-off point of monocytes to define the risk of mortality [25, 26]. Survival curves were estimated using the Kaplan–Meier method and compared by the log-rank test. Two-sided* P*<* * 0.05 was considered statistically significant for all statistical procedures.

## 3. Results

### 3.1. Patient Characteristics

Among the 121 patients with acute PQ poisoning from May 2012 to December 2018, 109 patients were enrolled in the study for further analysis, 7 patients presented incomplete data, 3 patients were not followed up, and 2 patients were transferred to another hospital. Among the 109 acute PQ poisoning patients, the survival rate was 41.28%. A total of 109 blood samples were obtained from patients to determine the PQ concentration and clinical laboratory examination upon arrival at the emergency room.  Table 1 shows that nonsurvivors exhibited a higher median creatinine of 104 mmol/L, plasma PQ concentration of 3.65 ng/mL, and monocyte of 0.72 ×10^9^/L in comparison with the survivors with creatinine of 62 mmol/L, plasma PQ concentration of 0.30 ng/mL, and monocyte of 0.36×10^9^/L.  Table 2 shows that the patients with elevated monocytes exhibited deteriorative creatinine (139 mg/dL vs. 72 mg/dL) and PaO_2_ (92.34 ±9.84 mmHg vs. 87.00 ±10.51 mmHg) and high plasma PQ concentration (7.80 ng/mL vs. 1.45 ng/mL).

### 3.2. Correlation Analysis

Correlation analysis showed that monocyte count positively correlated with plasma PQ concentration (r= 0.413;* P*< 0.001) and negatively correlated with survival time (r= 0.512;* P*< 0.001) and 90-day survival (r= 0.503;* P*< 0.001).

### 3.3. Cox Proportional Hazard Regression Analysis

In univariate analysis, plasma PQ concentration, creatinine, ALT, and monocyte count were selected from the Cox proportional hazard model for multivariate analysis (Table 3). Multivariate logistic regression analysis confirmed that plasma PQ concentration, creatinine, and monocyte count were independent risk factors for the 90-day survival of patients with acute PQ poisoning.

### 3.4. ROC Curve Analysis for 90-Day Mortality

**Figure 1:**
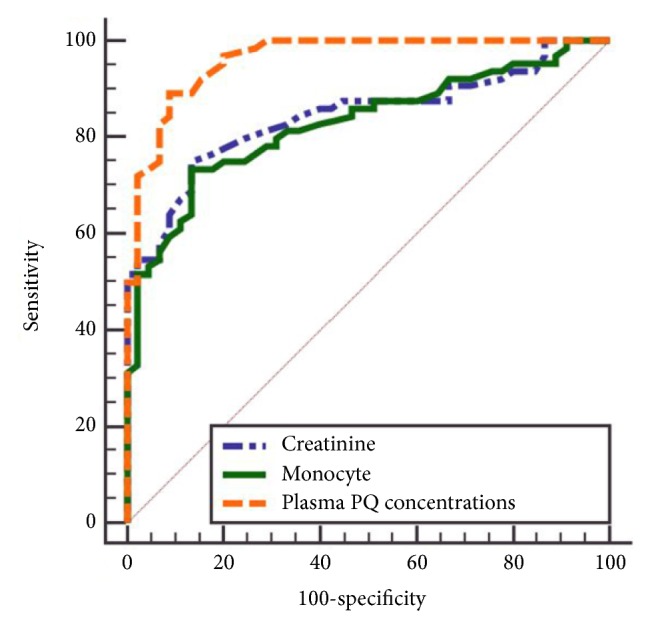
Area under the receiver operating characteristic curve analysis. PQ: paraquat.

The area under the curve for 90-day survival prediction was 0.962 of plasma PQ concentrations, 0.844 of creatinine, and 0.828 of monocyte count (Table 4 and Figure 1). Pairwise comparison showed that the predictive power of monocytes was lower than that of plasma PQ concentrations (*P* < 0.001) and similar to that of creatinine (*P* = 0.73). The optimal cut-off of monocyte count was 0.51×10^9^/L, and the sensitivity and specificity were 73.4% and 86.7%, respectively.

### 3.5. Kaplan–Meier Survival Analysis

**Figure 2:**
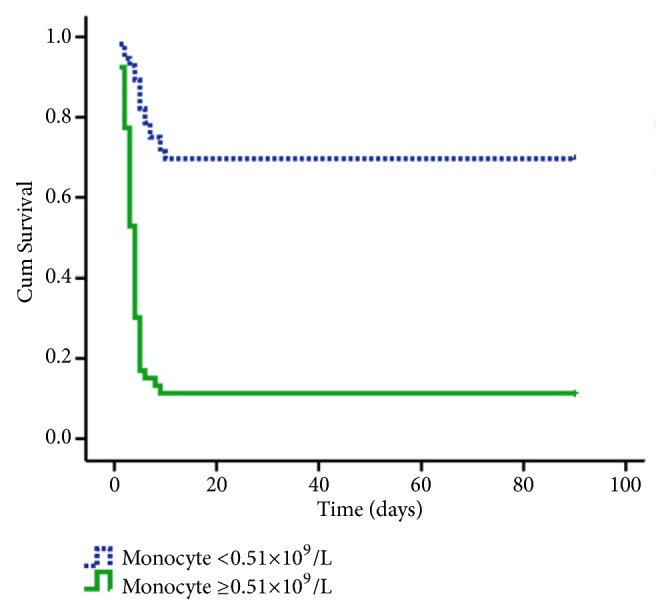
Kaplan–Meier analysis of survival curves for the groups according to monocyte count.

The Kaplan–Meier survival curve revealed that patients with elevated monocytes exhibited low 90-day survival (log-rank test;* P*< 0.001; Figure 2).

## 4. Discussion

This study is the first to specifically focus on the prognostic value of monocytes in patients with acute PQ poisoning. Our results show that elevated monocyte count upon patient admission is a predictive factor for 90-day survival. Furthermore, correlation analysis shows that monocytes are positively correlated with plasma PQ concentration and negatively correlated with survival time and 90-day survival.

Peripheral blood monocytes are a population of circulating mononuclear phagocytes that harbor potential to differentiate into macrophages and dendritic cells. After birth, monocytes derive from hematological precursors in the bone marrow and enter the blood circulation, from which they are recruited into tissues throughout the body. The monocyte chemoattractant protein-1 is a member of the C-C chemokine family and regulates the migration and infiltration of monocytes/macrophages. After exposure to PQ, bone marrow mesenchymal stem cells rapidly expressed monocyte chemotactic protein-1 in response to circulating toll-like receptor ligands induced by PQ and induced monocyte trafficking into the bloodstream [27, 28]. In addition to the bone marrow, a reservoir of splenic monocytes within the subcapsular red pulp can be recruited into the bloodstream according to CC chemokine receptor 2 [29–31]. Although the exact mechanism of monocytes is unclear, these studies pointed the way toward a detailed understanding and suggested further research directions.

As an important step for the onset and progression of PQ-induced lung injury, monocytes are recruited via chemotaxis such as monocyte chemoattractant protein-1, vascular cell adhesion molecule-1, and intracellular adhesion molecule 1, into the interstitial and alveolar spaces [32, 33]. Several potential mechanisms have been proposed for the associations between elevated monocyte and PQ-induced lung injury. First, activated monocytes migrating from the pulmonary vasculature into the interstitial and alveolar spaces enhance the production of chemokines and proinflammatory, such as tumor necrosis factor-*α* and IL-8, and activate the inflammatory system [21, 22, 34]. Second, monocyte-derived alveolar macrophages after their recruitment to the lung initiate an immune response and generate reactive oxygen species, leading to cellular NADPH depletion and lipid peroxidation of cell membranes [35]. Third, monocyte-derived alveolar macrophages increase the expression of transforming growth factor-*β*, which leads to robust profibrotic gene expression in fibroblasts, resulting in tissue fibrosis [36–38].

The primary target of toxicity in the lung is the alveolar epithelium. Exposure to PQ leads to the accumulation of PQ in the lungs, resulting in swelling, vacuolation, and disruption of mitochondria and the endoplasmic reticulum. This initial phase is followed by a proliferative phase where the alveolar space is filled with mononuclear profibroblasts that mature into fibroblasts within days to weeks. Previous studies showed that patients with PQ poisoning may be left with less diffusion dysfunction and restriction ventilation dysfunction at 2 to 3 months, and long-term follow-up suggests that respiratory function impairment usually fully recovers [39, 40]. High-resolution computed tomography revealed small cystic and linear shadows at the end of the first week, preponderant parenchymal abnormality after 2–4 weeks, focal honeycombing after 4 weeks, localized fibrosis containing small cysts at 9 months, and disappeared cystic changes at 1–2 years after PQ poisoning [41, 42].

The kidney is the main excretory organ of PQ, and PQ is predominantly excreted through the original form by the kidneys using glomerular filtration and active secretion. Thus, the kidneys are among the organs that can contain the highest concentration of PQ, which explains why remarkable acute renal injury occurs in the early stage of PQ intoxication. PQ is toxic to renal proximal tubule cells through the generation of reactive oxygen species, which cause lipid peroxidation of the cell membrane, leading to loss of membrane integrity and cell death. Other factors influencing the development of acute kidney injury include hypoperfusion from hypovolaemia and/or hypotension and direct glomerular injury [43]. The degree of kidney injury is confirmed as the important factor of prognosis [44–46].

Several limitations must be considered in the interpretation of the current results. First, as a retrospective study, this study has inherent information bias. Second, the sample size is too small because of the limited experimental conditions, hence the need for expansion or multicenter research. Third, monocyte count prior to PQ intoxication was not measured. Therefore, whether the elevated leucocyte levels were entirely due to PQ ingestion remains unconfirmed.

## 5. Conclusion

The present study demonstrated that elevated monocyte count is a useful early predictor of the 90-day survival of patients with acute PQ poisoning. When no facility measures PQ concentration, monocytes can be used as a reference for death risk in acute PQ poisoning.

## Figures and Tables

**Table 1:** General characteristics upon arrival between survival and mortality groups.

	Non-survival group (*n*=64)	Survival group (*n*=45)	*P* value
Age (years)	42.50 (31.75)	33.00 (21.00)	0.060
Gender (male/female)	27/37	20/25	0.815
Time from ingestion to gastric lavage (h)	1.00 (1.00)	1.00 (1.25)	0.627
Alanine aminotransferase (ALT, U/L)	32.65 (16.93)	26.60 (10.00)	0.006
Creatinine (*μ*mol/L)	104.00 (69.50)	62.00 (21.00)	<0.001
Alveolar oxygen partial pressure (PaO_2,_ mmHg)	89.82±10.50	93.90±12.85	0.050
Plasma PQ concentration (ng/mL)	3.65 (8.53)	0.30 (0.85)	<0.001
Monocyte (×10^9^/L)	0.72 (0.72)	0.36 (0.16)	<0.001

PQ: paraquat. Note. Continuous variables are presented as means ± SD or median (interquartile range) and categorical variable is presented as number.

**Table 2:** General characteristics upon arrival stratified according to monocyte count.

	Monocyte ≤1	Monocyte >1	*P* value
	(n=90)	(n=19)	
Age (years)	37.00 (27.00)	43.00 (28.00)	0.701
Gender (male/female)	40/50	7/12	0.543
Time from ingestion to gastric lavage (h)	1.00 (1.00)	1.00 (1.00)	0.617
Alanine aminotransferase (ALT, U/L)	28.15 (13.38)	32.50 (16.70)	0.088
Creatinine (*μ*mol/L)	72.00 (31.75)	139.00 (49.00)	<0.001
Alveolar oxygen partial pressure (PaO_2,_ mmHg)	92.34 ±9.84	87.00 ±10.51	0.036
Plasma PQ concentration (ng/mL)	1.45 (2.63)	7.80 (18.10)	<0.001

PQ: paraquat. Note. Continuous variables are presented as means ± SD or median (interquartile range) and categorical variable is presented as number.

**Table 3:** Cox regression model.

	Univariate COX model HR (95% CI)	*P* value	Multivariate COX model HR (95% CI)	*P* value
Age (years)	1.014 (1.000–1.029)	0.051	N/A	
Gender (male/female)	0.914 (0.557–1.502)	0.724	N/A	
Time from ingestion to gastric lavage	1.019 (0.859–1.209)	0.826	N/A	
Plasma PQ concentration	1.067 (1.048–1.087)	<0.001	1.026 (1.001–1.050)	0.038
Alanine aminotransferase (ALT)	1.010 (1.005–1.037)	0.010	1.015 (0.997–1.033)	0.109
Creatinine	1.019 (1.014–1.024)	<0.001	1.013 (1.006–1.019)	<0.001
Alveolar oxygen partial pressure (PaO_2,_)	0.978 (0.953–1.004)	0.101	N/A	
Monocyte	4.302 (2.684–6.897)	<0.001	1.841 (1.015–3.340)	0.044

N/A: not applicable; PQ: paraquat.

**Table 4:** ROC curve analysis.

Variable	Area under ROC curve	95% CI	Cutoff	Sensitivity (%)	Specificity (%)	Youden index
Plasma PQ concentration	0.962	0.930–0.994	1.55	89.1	91.1	0.802
Creatinine	0.844	0.771–0.917	74.5	75.0	86.7	0.617
Monocyte	0.828	0.751–0.904	0.51	73.4	86.7	0.601

PQ: paraquat; ROC: receiver operating characteristic; CI: confidence interval.

## Data Availability

The data used to support the findings of this study are included within the article.

## Abbreviations

PQ: Paraquat
AUC: Area under the curve
ROC: Receiver operating characteristic.

## References

[1] Wunnapuk K., Mohammed F., Gawarammana I. (2014). Prediction of paraquat exposure and toxicity in clinically ill poisoned patients: a model based approach. *British Journal of Clinical Pharmacology*.

[2] Gawarammana I., Buckley N. A., Mohamed F. (2018). High-dose immunosuppression to prevent death after paraquat self-poisoning – a randomised controlled trial. *Clinical Toxicology*.

[3] Wang Y., Chen Y., Mao L. (2017). Effects of hemoperfusion and continuous renal replacement therapy on patient survival following paraquat poisoning. *PLoS ONE*.

[4] Licker M., Schweizer A., Hohn L., Morel D. R., Spiliopoulos A. (1998). Single lung transplantation for adult respiratory distress syndrome after paraquat poisoning. *Thorax*.

[5] Elenga N., Merlin C., Le Guern R. (2018). Clinical features and prognosis of paraquat poisoning in French Guiana: A review of 62 cases. *Medicine*.

[6] Du Y., Mou Y. (2013). Predictive value of 3 methods in severity evaluation and prognosis of acute paraquat poisoning. *Journal of Central South University Medical sciences*.

[7] Scherrmann J. M., Houze P., Bismuth C., Bourdon R. (1987). Prognostic value of plasma and urine paraquat concentration. *Human & Experimental Toxicology*.

[8] Jones A. L., Elton R., Flanagan R. (1999). Multiple logistic regression analysis of plasma paraquat concentrations as a predictor of outcome in 375 cases of paraquat poisoning. *QJM: Monthly Journal of the Association of Physicians*.

[9] Proudfoot A. T., Stewart M. S., Levitt T., Widdop B. (1979). Paraquat poisoning: significance of plasma-paraquat concentrations. *Lancet*.

[10] Senarathna L., Eddleston M., Wilks M. (2009). Prediction of outcome after paraquat poisoning by measurement of the plasma paraquat concentration. *QJM: Monthly Journal of The Association of Physicians*.

[11] Li J., Zhao J., Zhang Q., Yuan F., Wei L. (2015). The value of assessment of area of ground glass opacity in lungs cast by high-resolution computed tomography on the prognosis of patients with acute paraquat intoxication. *Zhonghua Wei Zhong Bing Ji Jiu Yi Xue*.

[12] Liu J., Xiong Y., Jiang M. (2018). Ratio of injured lung volume fraction in prognosis evaluation of acute PQ poisoning. *BioMed Research International*.

[13] Kao C., Hsieh J., Ho Y., Hung D., Lin T., Ding H. (1999). Acute paraquat intoxication: using nuclear pulmonary studies to predict patient outcome. *CHEST*.

[14] Bao B., Li Z.-G., Sun X.-L. (2012). Blood lactic acid level and APACHE II score on prognosis of critically ill elderly patients. *Zhonghua Liuxingbingxue Zazhi*.

[15] Liu X., Ma T., Qu B., Ji Y., Liu Z. (2013). Prognostic value of initial arterial lactate level and lactate metabolic clearance rate in patients with acute paraquat poisoning. *The American Journal of Emergency Medicine*.

[16] Balın Ş. Ö., Tartar A. S., Akbulut A. (2018). The predictive role of haematological parameters in the diagnosis of osteoarticular brucellosis. *African Health Sciences*.

[17] Chiu Y. L., Shu K. H., Yang F. J., Chou T. Y., Chen P. M., Lay F. Y. (2018). A comprehensive characterization of aggravated aging-related changes in T lymphocytes and monocytes in end-stage renal disease: the iESRD study. *Immunity & Ageing : I & A*.

[18] Feng F., Zheng G., Wang Q. (2018). Low lymphocyte count and high monocyte count predicts poor prognosis of gastric cancer. *BMC Gastroenterology*.

[19] Li Z., Xu Z., Huang Y. (2018). The predictive value and the correlation of peripheral absolute monocyte count, tumor-associated macrophage and microvessel density in patients with colon cancer. *Medicine*.

[20] Moon J. M., Chun B. J., Cho Y. S., Lee S. M. Diagnostic Value of Parameters Related to White Blood Cell Counts for Troponin I Elevation in CO Poisoning.

[21] Liu Z., Wang Y., Zhao H., Zheng Q., Xiao L., Zhao M. (2014). CB2 receptor activation ameliorates the proinflammatory activity in acute lung injury induced by paraquat. *BioMed Research International*.

[22] Bianchi M., Bertini R., Fantuzzi G., Perin L., Salmona M., Ghezzi P. (1991). The pneumotoxicant paraquat induces IL-8 gene expression in human monocytes and pulmonary epithelial cells. *Cytokine*.

[23] George K. W., Chen A., Jain A. (2014). Correlation analysis of targeted proteins and metabolites to assess and engineer microbial isopentenol production. *Biotechnology and Bioengineering*.

[24] Romani A. A., Soliani P., Desenzani S., Borghetti A. F., Crafa P. (2006). The associated expression of Maspin and Bax proteins as a potential prognostic factor in intrahepatic cholangiocarcinoma. *BMC Cancer*.

[25] Yang X., Wu B., Ma S., Yin L., Wu M., Li A. (2018). Decreased expression of zwint is associated with poor prognosis in patients with HCC after surgery. *Technology in Cancer Research & Treatment*.

[26] Yang J., Guo X., Wu T., Niu K., Ma X. (2019). Prognostic significance of inflammation-based indexes in patients with stage III/IV colorectal cancer after adjuvant chemoradiotherapy. *Medicine*.

[27] Shi C., Jia T., Mendez-Ferrer S. (2011). Bone marrow mesenchymal stem and progenitor cells induce monocyte emigration in response to circulating toll-like receptor ligands. *Immunity*.

[28] Amirshahrokhi K., Khalili A.-R. (2016). Carvedilol attenuates paraquat-induced lung injury by inhibition of proinflammatory cytokines, chemokine MCP-1, NF-*κ*B activation and oxidative stress mediators. *Cytokine*.

[29] Paolillo N., Piccirilli S., Giardina E., Rispoli V., Colica C., Nisticò S. (2011). Effects of paraquat and capsaicin on the expression of genes related to inflammatory, immune responses and cell death in immortalized human HaCat keratinocytes. *International Journal of Immunopathology and Pharmacology*.

[30] Serbina N. V., Pamer E. G. (2006). Monocyte emigration from bone marrow during bacterial infection requires signals mediated by chemokine receptor CCR2. *Nature Immunology*.

[31] Tsou C.-L., Peters W., Si Y. (2007). Critical roles for CCR2 and MCP-3 in monocyte mobilization from bone marrow and recruitment to inflammatory sites. *The Journal of Clinical Investigation*.

[32] Bevilacqua M. P., Pober J. S., Mendrick D. L., Cotran R. S., Gimbrone M. A. (1987). Identification of an inducible endothelial-leukocyte adhesion molecule. *Proceedings of the National Acadamy of Sciences of the United States of America*.

[33] Li H., Cybulsky M. I., Gimbrone Jr M. A., Libby P. (1993). An atherogenic diet rapidly induces VCAM-1, a cytokine-regulatable mononuclear leukocyte adhesion molecule, in rabbit aortic endothelium. *Arteriosclerosis and Thrombosis : A Journal of Vascular Biology*.

[34] Dinis-Oliveira R. J., Sousa C., Remiao F. (2007). Sodium salicylate prevents paraquat-induced apoptosis in the rat lung. *Free Radical Biology & Medicine*.

[35] Yeh S. T., Guo H., Su Y. (2006). Protective effects of N-acetylcysteine treatment post acute paraquat intoxication in rats and in human lung epithelial cells. *Toxicology*.

[36] He C., Murthy S., McCormick M. L., Spitz D. R., Ryan A. J., Carter A. B. (2011). Mitochondrial Cu,Zn-superoxide dismutase mediates pulmonary fibrosis by augmenting H2O2 generation. *The Journal of Biological Chemistry*.

[37] Jain M., Rivera S., Monclus E. A. (2013). Mitochondrial reactive oxygen species regulate transforming growth factor-*β* signaling. *The Journal of Biological Chemistry*.

[38] Gibbons M. A., MacKinnon A. C., Ramachandran P. (2011). Ly6Chi monocytes direct alternatively activated profibrotic macrophage regulation of lung fibrosis. *American Journal of Respiratory and Critical Care Medicine*.

[39] Lin J., Liu L., Leu M. (1995). Recovery of respiratory function in survivors with paraquat intoxication. *Archives of Environmental Health: An International Journal*.

[40] Gao J., Cao Z., Feng S. (2018). Patients with mild paraquat poisoning treated with prolonged low-dose methylprednisolone have better lung function: A retrospective analysis. *Medicine*.

[41] Im J.-G., Lee K. S., Han M. C., Kim S. J., Kim I. O. (1991). Paraquat poisoning: Findings on chest radiography and CT in 42 patients. *American Journal of Roentgenology*.

[42] Lee K., GIL H., Kim Y., Yang J., Lee E., Hong S. (2009). Marked recovery from paraquat-induced lung injury during long-term follow-up. *The Korean Journal of Internal Medicine*.

[43] Chan B. S. H., Lazzaro V. A., Seale J. P., Duggin G. G. (1998). The renal excretory mechanisms and the role of organic cations in modulating the renal handling of paraquat. *Pharmacology & Therapeutics*.

[44] Ragoucy-Sengler C., Pileire B. (1996). A biological index to predict patient outcome in paraquat poisoning. *Human & Experimental Toxicology*.

[45] Hong S., Yang D., Hwang K. (2000). Associations between laboratory parameters and outcome of paraquat poisoning. *Toxicology Letters*.

[46] Roberts D. M., Wilks M. F., Roberts M. S. (2011). Changes in the concentrations of creatinine, cystatin C and NGAL in patients with acute paraquat self-poisoning. *Toxicology Letters*.

